# Natural Kelp (*Laminaria japonica*) Hydrogel with Anisotropic Mechanical Properties, Low Friction and Self-Cleaning for Triboelectric Nanogenerator

**DOI:** 10.3390/gels11080597

**Published:** 2025-08-01

**Authors:** Dongnian Chen, Hui Yu, Jiajia Hao, Qiang Chen, Lin Zhu

**Affiliations:** 1 Taizhou Institute of Science and Technology, Nanjing University of Science and Technology, Taizhou 225300, China; 22007010@nustti.edu.cn; 2 Wenzhou Institute, University of Chinese Academy of Sciences, Wenzhou 352001, China; yuhuier_2008@163.com (H.Y.); haojia20210309@163.com (J.H.); 3 Oujiang Lab, Wenzhou 352001, China

**Keywords:** kelp, anisotropic, low friction, self-cleaning, triboelectric nanogenerators

## Abstract

Kelp is a natural hydrogel material, which has been widely used in food industry. However, as a natural material, its properties have not been well explored. In this work, the surface and mechanical properties of kelp were investigated. The surface of kelp exhibited superoleophobicity and a self-clean property. The friction coefficient (COF) of the kelp surface was also low (<0.1). Interestingly, kelp demonstrated anisotropic mechanical properties either with or without metal ions. The tensile strength and toughness of kelp along with the growth direction (H) were better than those at the direction vertical to the growth direction (V). The adsorption of metal ions would significantly enhance the mechanical properties and ionic conductivity. Triboelectric nanogenerator (TENG) was assembled using kelp with NaCl, which showed excellent output performance (open-circuit voltage of 30 V, short-circuit current of 0.73 μA and charge transfer on contact of 10.5 nC). A writing tablet was prepared to use as the kelp-based self-powered tactile sensor. This work provides a new insight into natural kelp, which may be used as a renewable material.

## 1. Introduction

*Laminaria japonica*, commonly known as kelp or seaweed, is a brown macroalga with a complex hierarchical structure adapted to thrive in intertidal and subtidal zones [1]. Kelp is widely distributed in cold and temperate coastal waters, plays a significant role in marine ecosystems and has great value for humans [2]. Kelp has a unique structure composed of a holdfast, stipe and blades. The holdfast, anchor-like, secures the kelp to the seabed, providing stability and enabling it to withstand ocean currents and waves [3]. The stipe, resembling a stem, connects the holdfast to the blades and serves as the main support structure, helping the blades extend toward the water’s surface to access sunlight for photosynthesis. The blades, the largest part of the kelp, are flat and leaf-like, and are the primary sites for photosynthesis and nutrient absorption. They are often broad and long, with some species’ blades reaching several meters in length [4].

At the cellular level, the blade features a multilayered architecture: (1) an outer epidermal layer coated with a gelatinous polysaccharide-rich matrix (primarily alginates and fucoidans) that provides protection and lubrication; (2) a middle cortical layer containing densely packed parenchyma cells reinforced by cellulose microfibrils and hemicellulose networks; (3) an inner medullary layer composed of elongated tubular cells and mucilage-filled channels, which facilitate nutrient transport and structural flexibility [5,6]. The composition of seaweed is dominated by alginate (a linear copolymer of guluronic and mannuronic acids), cellulose, fucoidan (sulfated polysaccharides) and laminarin (a storage glucan), all contributing to its unique biomechanical and biochemical properties [7]. Among them, alginate can form a viscous colloidal solution or gel in water, giving kelp its slippery texture and making it useful in food processing and other industries [8]. Mannitol has a sweet taste and acts as a natural sweetener and moisturizer [9]. Kelp possesses a self-lubricating property, primarily attributed to the alginic acid and mannitol [10]. Alginate can absorb water and form a slippery hydrogel layer containing mannitol on the surface of the kelp, reducing friction between surfaces [11]. This self-lubricating property allows kelp to minimize friction and wear in water, facilitating its movement and growth. It also helps kelp adapt to the fluid marine environment and reduces energy consumption.

Due to its growth environment and mode, kelp exhibits anisotropy. During growth, kelp is subjected to ocean currents, waves and other external forces [12]. The side facing the current experiences greater mechanical stress and stimulation, leading to differences in structural and mechanical properties compared to the leeward side [13]. For example, the cells on the windward side may be more densely packed and have thicker cell walls, resulting in higher tensile strength and stiffness [14]. The blades may also display anisotropy. Their longitudinal and transverse mechanical properties should differ significantly. However, the anisotropic mechanical properties are rarely investigated [15].

**Figure 1:**
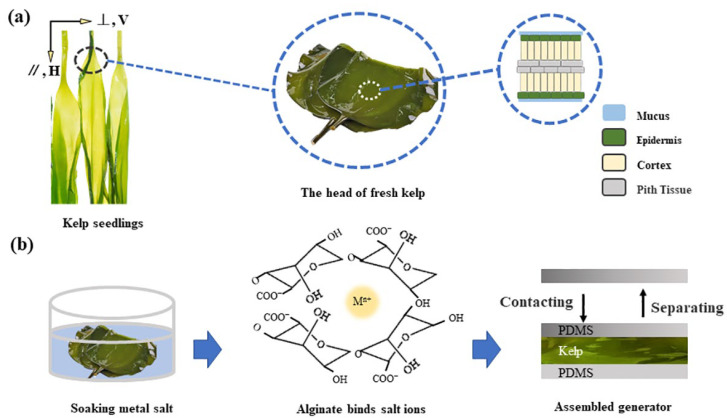
(**a**) The location and morphological structure of the kelp samples. (**b**) Kelp modification methods, principles and applications.

In this paper, the surface properties of natural kelp were investigated firstly and then the anisotropic mechanical properties were studied. As shown in Figure 1a, the growth direction was defined as (//, H) and the direction vertical to the growth direction was defined as (⟂, V) [16]. All the samples near the stipe were selected and the tensile strength, tear properties and toughness of the kelp in the H and V-directions were tested. The dry content of alginate in kelp is about 24%, which can be chelated with metal ions [17]. As shown in Figure 1b, we also exploited this property to explore the changes in the structure and strength of kelp after soaking in metal ion solution. Finally, triboelectric nanogenerator (TENG) was assembled using kelp adsorbed with persalt ions. Due to the special surface properties of the material during the working process, the assembled TENGs had lower energy loss, thereby increasing the output power. In addition, this renewable natural material could greatly alleviate the problem of material vacancies and promote the development of material applications.

## 2. Results and Discussion

### 2.1. Surface Properties

**Figure 2:**
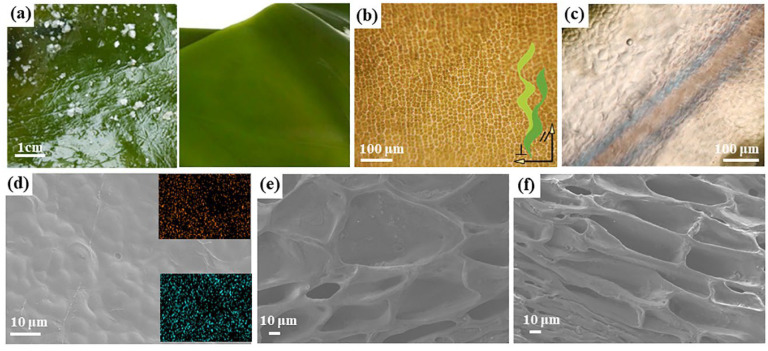
Morphological characteristics of kelp (**a**) purchased raw kelp and washed kelp. (**b**,**c**) Surface and section optical photographs of kelp. (**d**) SEM image of the kelp surface, with the distribution of iodine and potassium elements from top to bottom. (**e**,**f**) SEM images of the V and H-directions of the kelp section.

There are a lot of kelps cultured in the coastal areas in China [18]. After the whole kelp was salvaged and cleaned, it was salted to form pickled kelp for long-time storage. Figure 2a on the left was a photo of the salted kelp purchased in this work and on the right was a photo of simply washing off the surface salt particles. In order to exclude other inorganic salts from affecting the properties of kelp, the kelp was firstly desalination treatment. After 6 h of immersion in water, the Cl^−^ ions in the pickled kelp were basically completely removed and the weight and thickness of the kelp did not increase significantly (Figure S1a). As a natural material, kelp’s macroscopic mechanical performance was closely related to the microstructures. In Figure 2b, it was found that there were folding and protruding mound-like structures on the surface and they were evenly arranged in blocks along the H-direction, with strong orientation. As shown in the model in Figure 1, the epidermal cells were small, neat and compact, protecting the surface of kelp. In the optical image of the cross-section of kelp, dense meristematic and cortical areas could be detected, and the epidermis, cortex and medulla were clearly distinguished (Figure 2c). The presence of hemispherical hillocks in the epidermis could also be seen from the SEM photo (Figure 2d). EDS proved that the kelp surface was uniformly and densely distributed with elements rich in iodine and potassium. The cortex was below the surface cells, which contained more glia and water. After freeze-drying, the water evaporated to reveal overlapping cell voids (Figure S2). At the same length, the cell cross-section in the V-direction (Figure 2e) was wider than that in the H-direction (Figure 2f). Kelp contains a lot of water and it has a three-dimensional network structure after dehydration, which is a natural hydrogel. This structure enables kelp to grow under the impact of waves.

**Figure 3:**
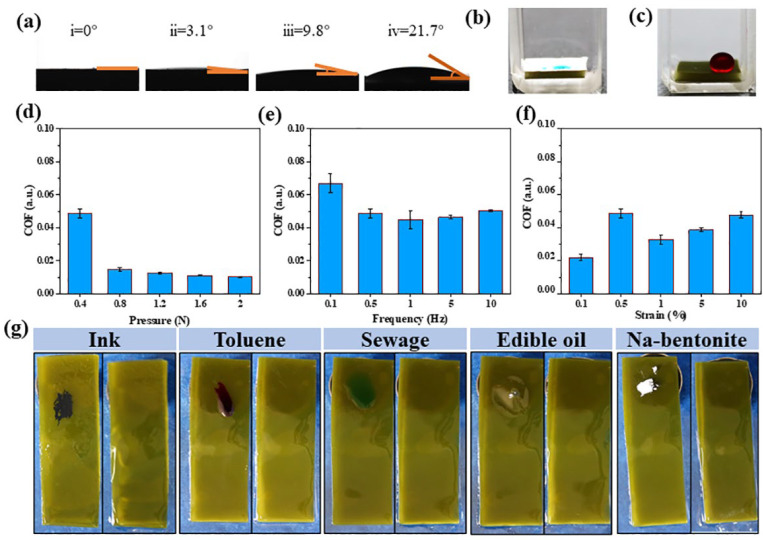
(**a**) 1–4 are the static contact angles of water, toluene, silicone oil, edible oil and kelp surface in air medium, respectively. (**b**) Water contact angle in edible oil medium, (**c**) photo demonstration of chloroform (red oil O staining) contact angle in aqueous medium. The friction coefficient of a kelp surface is related to (**d**) normal pressure, (**e**) shear frequency and (**f**) shear strain. Comparison standard: normal pressure 0.4 N, frequency 0.5 Hz, strain 0.5%. (**g**) The surface of the kelp was resistant to ink, toluene, dirty water, edible oil and Na-bentonite.

There were sporangia in the lower part of the leaves of kelp, as shown in Figure 3c, with a mucus cavity, which could secrete slippery substances, making it difficult for fouling microorganisms to adhere. In Figure 3a, the static contact angles (CA) of water, toluene, silicone oil, edible oil and the kelp surface in air were measured. The results showed that the water contact angle (WCA) in air was close to 0°. The surface of natural kelp is super-hydrophilic, which is caused by the rich in hydrophilic groups such as hydroxyl and carboxyl groups [19]. Surprisingly, the contact angles of toluene, silicone oil and edible oil were only 3.1°, 9.8° and 21.7°, respectively. Due to the presence of mannitol on the surface and microscopic rough structure, the surface of kelp exhibits excellent wettability to non-polar/weakly polar liquids with low surface tension such as toluene, silicone oil and edible oil, resulting in a low contact angle. The underwater contact angle of kelp in oil remained at 0° (Figure 3b), but the oil contact angle (OCA) underwater was as high as 157° (Figure 3c) and the oil droplets rolled off quickly as the kelp was slightly tilted (less than 4°), indicating that the oil droplets could not adhere to the surface. The results indicate that the kelp surface possesses underwater superoleophobicity properties. The water content of natural kelp was as high as 93.8 ± 0.2%. Under the applied pressure, the dome-shaped structure can be deformed, thus resisting part of the elastic force. Meanwhile, the mucus is attached to the surface of the kelp [20]. The combination of the two will make the kelp have good lubricating properties. The effects of normal pressure, shear frequency, and shear strain on the coefficient of friction (COF) of kelp were explored. As shown in Figure 3d, with the increase of positive pressure, the COF decreases, from 0.048 to 0.01. It was further proved that the elastic mechanism of the mound-shaped structure enabled the kelp surface to buffer part of the pressure. From the COF data in Figure 3e,f, there was no direct linear relationship between the frequency and amplitude, while all the COFs were lower than 0.08. The self-cleaning properties of the kelp surface were also explored. In Figure 3g, ink, toluene (red oil O dyeing), sewage (olive green dyeing), edible oil and Na-bentonite were used to decontaminate the kelp for 1 min and then it was rinsed with water. All the contaminants were easily rinsed off and the kelp was maintained with the cleanliness of the original surface. When the kelp was slightly tilted (less than 4°), the oil droplets rolled off quickly. The ultra-lubricated surface of kelp makes it difficult for contaminants to adhere to it, showing a self-cleaning property.

### 2.2. Mechanical Properties

**Figure 4:**
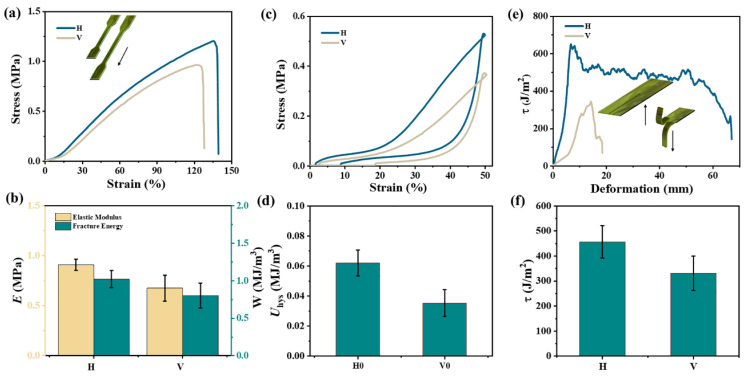
After repeated dipping of kelp for 6 h, the tensile curves of the samples in the H-direction and V-direction (**a**,**b**), elastic modulus and fracture toughness, (**c**,**d**) loading-unloading stress-strain curves and dissipated energy, (**e**,**f**) relationship between tearing energy and displacement, average tearing energy.

Due to its harsh marine environment, natural kelp must have superior mechanical properties to survive [21]. Pickled kelp is suitable for storage and does not affect the nutritional content of fresh kelp [22]. Before exploring the mechanical strength of kelp, it was necessary to clarify the relationship between the strength of kelp and its thickness. The thickness of kelp was selected in the interval of 0.5~3.5 mm and it was stretched along the H-direction. As shown in Figure S1c, there was no linear correlation between the elongation and breaking strength and the thickness of kelp. As a naturally grown material, kelp has fiber orientation and the H and V-directions will show different mechanical properties. The stress-strain curve of the uniaxial tensile test is shown in Figure 4a. The tensile strength (*σ*_f_) and tensile strain at break (*ε*_f_) of H were 1.20 MPa and 135%, respectively, which were all greater than those of the V-direction (0.96 MPa and 123%). Consistently, the data in Figure 4b also showed that the elastic modulus (*E*) and work of extension (*W*) in the H-direction were 0.91 MPa and 1.02 MJ/m^3^, respectively, which were greater than those of the V-direction (0.67 MPa and 0.80 MJ/m^3^). The dissipative properties of kelp were evaluated by loading-unloading curves. As shown in Figure 4c, at a fixed strain of 50%, the hysteresis loop of H-direction was also larger than that of V-direction. The dissipated energy in the H-direction was 0.06 MJ/m^3^, which was higher than the V-direction (0.03 MJ/m^3^) (Figure 4d). Figure 4e examined the tear properties of kelp, and the “trouser-shaped” samples were torn along the H and V-directions, respectively. The tearing curves were obviously different. A steady tearing of H-direction kelp was achieved and the tearing energy was 456 J/m^2^. However, for V-direction kelp, when the maximum tearing force was achieved, the kelp was fractured rapidly. The tearing energy of V-direction kelp was 331 J/m^2^, which was also weaker than that of H-direction kelp (Figure 4f). In the evidence, kelp illustrates distinct anisotropic mechanical properties.

**Figure 5:**
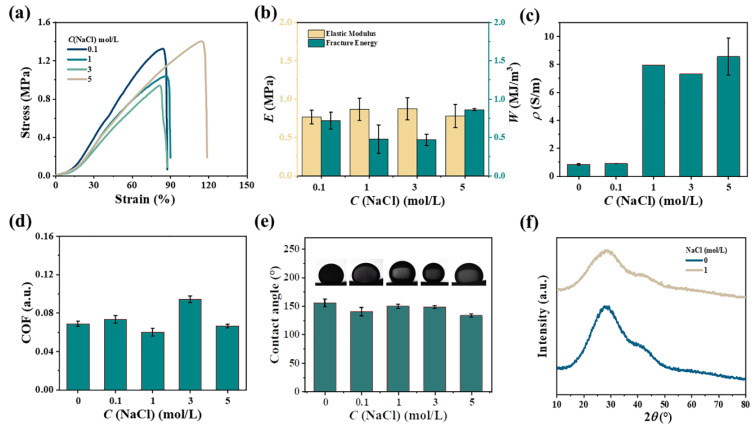
(**a**) Kelp tensile curve after soaking in NaCl solution. (**b**) Elastic modulus and fracture toughness. (**c**) Conductivity. (**d**) COF (surface friction coefficient). (**e**) Underwanter static contact angle of oil (chloroform CHCl_3_). (**f**) XRD curves of washed and soaked kelp with 1 mol/L NaCl.

Algae have a strong ability to enrich a variety of metal ions and have great development prospects for industrial metal wastewater purification [23,24]. The mechanical properties of kelp adsorbed with metal ions changed accordingly. Figure 5a showed the tensile stress-strain curves of kelp along the H-direction after soaking in NaCl solutions with different concentrations. When the NaCl concentration increased from 0.1 to 3 mol/L, *σ*_f_ of kelp decreased slightly from 1.32 to 0.93 MPa and *ε*_f_ changed little from 83 to 81%. Further increases to 5 mol/L, *σ*_f_ and *ε*_f_ were to 1.4 MPa and 114%, respectively. The concentration of NaCl had a negligible effect on the elastic modulus of kelp, which was stable at 0.82 ± 0.05 MPa (Figure 5b). Overall, the tensile properties of kelp were little affected by NaCl solutions. Kelp contains a large amount of water and conductive ions and even without additional adsorption of salt ions, it had an ionic conductivity of 0.85 S/m. The ionic conductivity was greatly improved after soaking in NaCl solution. As shown in Figure 5c, the concentration of NaCl increased from 0.1 to 5 mol/L and the ionic conductivity of kelp increased from 0.91 to 8.56 S/m. At the same time, the presence of NaCl did not change the low friction characteristic of the kelp; all the friction coefficients at various NaCl concentrations were lower than 0.1. More interestingly, the NaCl solutions had no effect on the superoleophobic properties of the kelp, and the underwater OCA was maintained at 145 ± 8°. In Figure 5f, X-ray Diffraction (XRD) data showed that kelp or kelp with NaCl solutions exhibit weak crystalline structure.

### 2.3. Enhancement with Various Metal Ions

**Figure 6:**
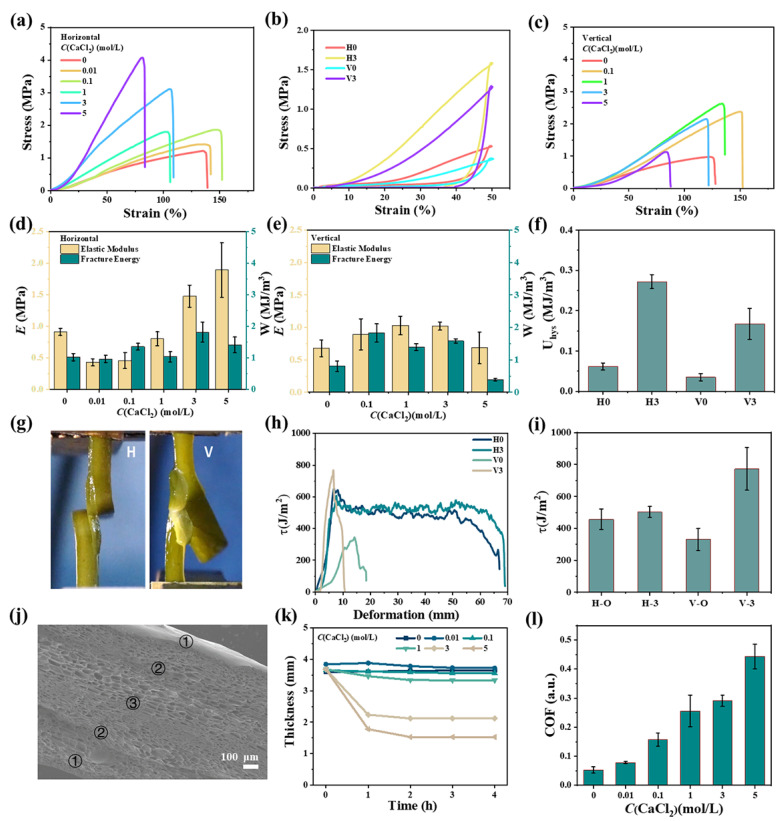
(**a**) Stretching in the H-direction of kelp, stress-strain relationship between CaCl_2_ solution and kelp, (**b**) histogram of elastic modulus and fracture toughness. (**c**) V-direction stretching, stress-strain curves of CaCl_2_ solution and kelp and (**d**) bar graphs of elastic modulus and fracture toughness. (**e**) Load-unload stress-strain curve. (**f**) Energy dissipated. (**g**) Photographs of the tear in the H and V-directions. (**h**) Tear versus displacement curves. (**i**) Tear energy. (**j**) SEM image of the cross-section in the V-direction after kelp foam with CaCl_2_, (**k**) the relationship between soaking time and thickness. (**l**) The relationship between the coefficient of friction (COF) of the kelp surface and the salt concentration.

Alginate solutions are in the cell walls of Laminariales. Metal ions can form coordination bonds with carboxyl groups in kelp, which will improve their mechanical properties, water resistance and rigidity. Figure 6a shows the stress-strain curves of kelp soaked in CaCl_2_ solutions. When the concentration of CaCl_2_ increased from 0 to 3 mol/L, *σ*_f_ of kelp in the H-direction increased from 0.90 to 3.14 MPa. However, *ε*_f_ did not change significantly and was around 100%. The data in Figure 6b show that *E* and *W* also increased from 0.62 to 1.47 MPa and from 0.57 to 1.81 MJ/m^3^, respectively. However, when the concentration of CaCl_2_ exceeded 3 mol/L, although the fracture strength was increased to 3.86 MPa, other mechanical properties were weakened and the kelp became particularly brittle and it tore as soon as it was broken. Plant materials usually contain anisotropic structures. Figure 6c indicated that *σ*_f_ of kelp increased from 1.32 to 2.52 MPa in the V-direction, which was not much higher than that in the H-direction. At the same time, *ε*_f_ did not change significantly, about 120%. The *E* and *W* also increased from 1.32 to 2.51 MPa and from 0.70 to 1.53 MJ/m^3^, respectively. Fallaciously, when the concentration of CaCl_2_ was greater than 3 mol/L, other mechanical properties were weakened. Cyclic loading-unloading tests were performed to illustrate the energy dissipation of kelp. The loading curve in Figure 6e did not overlap with the unloading curve and a hysteresis loop was observed. When loaded to the same strain and then unloaded, the dissipated energy (*U*_hys_) in the H-direction was 0.06 MJ/m^3^, which was larger than that in the V-direction (0.03 MJ/m^3^). After soaking in CaCl_2_ solution, all the hysteresis loops became larger (Figure 6f). Consistently, *U*_hys_ in the H-direction was 0.27 MJ/m^3^, which was still larger than 0.16 MJ/m^3^ in the V-direction. Because of the fiber orientation of kelp in the H-direction, when the sample was stretched, the force received during the stretching in the H-direction was very stable, but after reaching the maximum tear value in the V-direction, it was immediately torn along the fiber direction. The phenomenon was the same as the sample without salt and the photos of the tearing process were shown in Figure 6g. Further, the tearing curves were illustrated in Figure 6h and it was proved that CaCl_2_ did not have a great influence on the fiber structure of kelp and the tearing energy in the H-direction was maintained at 475 ± 25 J/m^2^. In contrast, the tearing in the V-direction had been greatly improved, from 331 J/m^2^ to 772 J/m^2^. Compared with the SEM images of un-adsorbed Ca^2+^ ions (Figure 2e,f), the SEM in Figure 6j could clearly see the epidermal tissue, cortical tissue and medullary tissue of kelp and the thickness was thinner because the diameter of the shrinkage hole was reduced. To further illustrate this phenomenon, after soaking in different concentrations of CaCl_2_ salt solution for 2 h, the change in the weight of kelp was recorded. As shown in Figure 6k, when the concentration was 1 mol/L or below, the weight remained above 90%. When the salt concentration increased to 3 mol/L and 5 mol/L, a large amount of water in the kelp was displaced due to the large osmotic pressure difference, so that the weight of the kelp decreased and reached equilibrium after 2 h. The friction of the contact material was a complex process. It could be seen from Figure 6l that the excellent performance of kelp with low friction cannot be maintained after immersion in Ca^2+^ solution, indicating that the high-concentration salt solution destroys the polysaccharide structure secreted on the surface of kelp.

**Figure 7:**
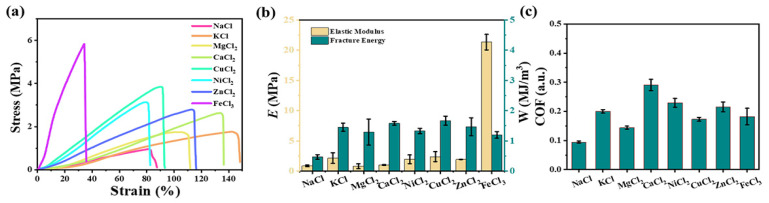
(**a**) Tensile curves of salt species and kelp, (**b**) relationship between tensile modulus and fracture toughness. (**c**) Coefficient of friction (COF) on the surface of kelp.

The metal ions adsorption mechanism of alginate is mainly based on ion exchange and the adsorption capacity of different metals is different. In order to explore the characteristics of kelp’s response to various metal ions, seven chloride salts were compared in this work. As shown in Figure 7a, after soaking in monovalent KCl, the mechanical properties of kelp were enhanced, corresponding to *σ*_f_ ~1.89 MPa and *ε*_f_ ~143%, although the enhancement mechanism of K^+^ ions for kelp is still perplexing. In divalent MgCl_2_, CaCl_2_, NiCl_2_, CuCl_2_ and ZnCl_2_, with the increase of molecular weight, the fracture strength of kelp gradually increased and the maximum strain gradually decreased. It could be seen from Figure 7b that divalent metal ions had little effect on the fracture toughness of kelp, but had a greater effect on its elastic modulus. In 3 mol/L FeCl_3_ solution, E of kelp was as high as 21 MPa. Figure 7c showed that the friction coefficient of the kelp in various metal ion solutions was increased compared to NaCl. The results indicate the mechanical properties of kelp can enhance by various metal ions.

### 2.4. Properties of TENG

**Figure 8:**
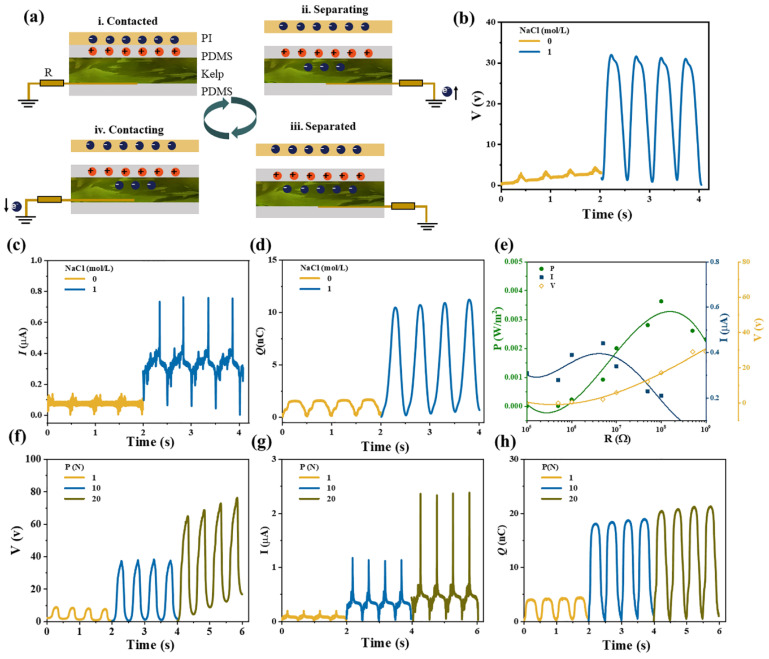
(**a**) The working principle of kelp used as single-electrode triboelectric power generation. (**b**) Voltage, (**c**) current, (**d**) electric capacity and (**e**) output power per unit area. The relationship between the normal positive pressure (P) applied during the rubbing process of the assembled device and the output (**f**) voltage, (**g**) current, (**h**) capacitance.

Triboelectric nanogenerators (TENGs) [25], as an energy supply unit and a self-powered sensor unit, can effectively convert various forms of environmental mechanical energy into electrical energy by utilizing the coupling effect of contact electrification and electrostatic induction [26], representing a promising potential for applications. The development of new triboelectric materials is of great significance for extending the current triboelectric family. In this work, natural kelp was used to fabricate TENG sensors [27,28]. Polyimide (PI) was designed as a negative triboelectric material with an effective contact area of 10 cm^2^ and polydimethylsiloxane (PDMS) was used as a positive triboelectric and packaging material, which was connected to the ground through kelp and wires to form a single-electrode device. In the pristine state, PI and PDMS were in contact and no charge was generated or induced (Figure 8(ai)). When PI and PDMS were separated, a potential difference was formed between the kelp and PDMS and the flow of electrons from the ground to the kelp would generate an electrical signal (Figure 8(aii)). Positive charges were left on PDMS and negative charges were left on PI. When PI and PDMS were completely separated, the induced positive charge in PDMS could balance the negative charge on kelp, resulting in a zero signal in this state (Figure 8(aiii)). When the PI contacted PDMS again, the induced negative charge in the kelp flows to the ground to generate an opposite electrical signal (Figure 8(aiv)). Until the two surfaces touched again, the electrostatically induced charge was lost and a zero electrical signal appeared. Based on the model in Figure 8a, the single-electrode mode SC-TENG was assembled. The output performance before and after adsorption of 1 M NaCl was investigated (Figure 8b–d). After adsorption of sodium ions, the open circuit voltage, short circuit current and the charge transfer amount at the time of contact were 30 V, 0.73 μA and 10.5 nC, respectively, which were much higher than those of without Na^+^ ions (2 V, 0.12 μA and 1.4 nC). As the resistance increased, the current decreased and the voltage increased, the output power per unit area first increased and then decreased, and the maximum output power was 3.63 mW/m^2^. The effect of normal force on output performance was also investigated (Figure 8e,f). The output performance was 37 V, 1.1 μA and 18.3 nC when subjected to a normal force of 10 N, which was 4 times that of a normal force of 1 N (8 V, 0.17 μA and 4.1 nC). When the normal force was increased to 20 N, the open circuit voltage was increased to 64V and the output current was also increased to 2.3 μA. The amount of charge transfer was also increased to 21 nC.

**Figure 9:**
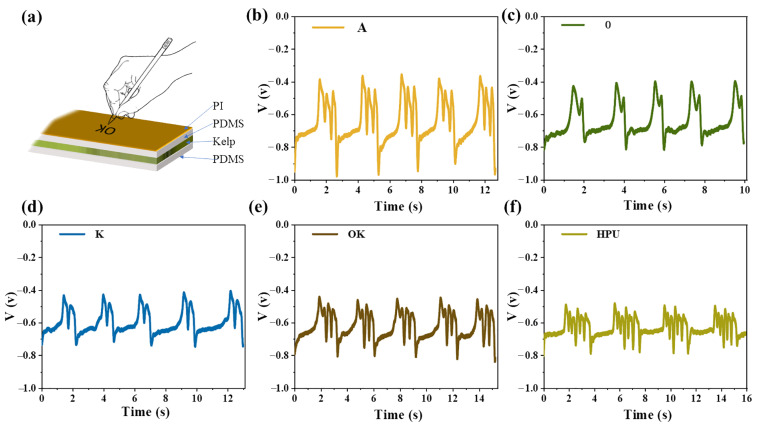
(**a**) Used a capacitive pen to write on the device assembled with kelp. (**b**–**f**) Wrote the continuous voltage output of “A, B, O, K, OK”.

To further broaden the application of kelp-based SC-TENG, a writing tablet was prepared to use as the self-powered tactile sensor [29,30]. Figure 9a showed a schematic diagram of the device used for high-sensitivity handwriting recognition, where the pen used was PVC plastic. TENGs could generate special voltage signals with different peak shapes and numbers according to the pressure of the pen tip, corresponding to several representative letters (“A” (Figure 9b), “B” (Figure 9c), “O” (Figure 9d), “K” (Figure 9e)) or the word “OK” (Figure 9f). Writing on the surface of the TENG was repeatable, where each signal group represents a complete handwriting, and the output voltage of the TENG was highly repeatable when the same content is written by a particular author [31]. Therefore, kelp-based TENGs had high sensitivity, reliability, and recognizability in the application of self-powered tactile sensors [32]. In the future, after machine learning and subsequent processing, the signal was expected to serve as a self-powered written text recognizer and an encryptor of confidential information.

## 3. Conclusions

In summary, natural kelp hydrogels exhibit remarkable surface and mechanical properties, as evidenced by microscopic observations, friction tests and mechanical assessments. The surface of kelp demonstrates underwater superoleophobicity, characterized by minimal contact angles for both water and oil, endowing it with self-cleaning capabilities. This property is attributed to the unique mound-like surface structure of kelp, which also contributes to its low coefficient of friction (COF < 0.1). Mechanically, kelp displays anisotropic behavior, with superior tensile strength and toughness along the growth direction (H) compared to the vertical direction (V). The adsorption of metal ions significantly enhances both the mechanical properties and ionic conductivity of kelp hydrogels. Specifically, while NaCl has a negligible effect on mechanical properties, it increases ionic conductivity to 8.56 S/m. In contrast, adsorption of Ca^2+^ ions markedly boosts fracture strength to 3.86 MPa and Fe^3+^ ions elevate the elastic modulus to 21 MPa. Furthermore, kelp hydrogels can be utilized to assemble triboelectric nanogenerators (TENGs) for self-powered sensing devices. The TENGs exhibit exceptional energy output, with a maximum power of 3.63 mW/m^2^. Upon adsorption of 1 mol/L Na^+^, the open-circuit voltage reaches 30 V, the short-circuit current is 0.73 μA and the induced charge transfer is 10.5 nC. These TENGs, configured as tablets, can reliably transmit information from specific writing content, showcasing ultra-high sensitivity to mechanical stimuli. This sensitivity holds significant potential for applications in self-powered monitoring and high-precision stroke recognition. Kelp hydrogels, renewable and biodegradable, offer scalability and environmental benefits, making them ideal for large-scale applications in packaging, textiles and biomedicine. Their self-cleaning, low-friction and energy-harvesting properties highlight potential for bio-based sensors and energy devices. Future research should optimize these properties for specific uses, such as improving kelp-based TENGs’ durability and conductivity, and developing synergistic composite materials.

## 4. Materials and Methods

### 4.1. Materials

Kelp was purchased from Rongcheng Xinwen breeding Co., Ltd. (Rongcheng, China), in the Bohai sea of China. NaCl (A.R.), CaCl_2_ (A.R.), FeCl_3_ (A.R.), KCl (A.R.), CuCl_2_ (G.R.) and NiCl_2_ (A.R.) were obtained from Aladdin Biochemical Technology Co., Ltd. (Shanghai, China). K_2_CrO_4_ (A.R.), MgCl_2_ (A.R.) and ZnCl_2_ (A.R.) were accepted by Tianjin Kermel Chemical Reagent Technology (Tianjin, China).

### 4.2. Kelp Pretreatment

#### 4.2.1. Removal of Cl^−^ in Salted Kelp

Since we used salted kelp during the experiment, we immersed it in water to reduce the salt concentration in order to restore its properties as much as possible. The water solution completely covered the kelp, changing the water every 2 h. We took salt water, then measured Cl^−^ concentration before changing water. The end point of titration was determined by Mohr method. The restored kelp was ready, which was used to adsorb metal ions.

#### 4.2.2. Introducing Metal Ion Mn^+^ into Kelp

A salt solution of a certain concentration was prepared in advance. The restored kelp was soaked in a salt solution in a fixed proportion with about 40 g kelp /100 mL salt solution. The parameters of kelp were recorded every 2 h.

### 4.3. Testing and Characterization

#### 4.3.1. Optical Microstructure Characterization

The microscopic topography of the surface and cross-section of the kelp samples was observed using a SMART-POL polarizing microscope (China). The surface and cross-sectional morphology images were taken with a Canon EOS 750D camera (Janpan).

#### 4.3.2. Scanning Electron Microscopy (SEM) Structural Characterization

The microstructure of the hydrogels was characterized using a Zeiss Merlin Compact field emission scanning electron microscope (Germany). The soaked kelp samples were quenched with liquid nitrogen and further completely dried in a freeze-dryer. Then, select the cross-sectional surface for gold spraying for 40 s and then prepare it for later use. The morphology and structure of the samples were observed at different magnifications at a voltage of 15 kV.

The samples were subjected to surface energy dispersive spectroscopy (EDS) analysis using Merlin Compact field emission electron scanning electron microscope from Zeiss, Germany, to explore the element species and their distribution on the surface.

#### 4.3.3. Coefficient of Friction (COF)

The tribological properties of kelp samples were tested using an advanced rotational rheometer (Anton Paar Trading Co., Ltd., Austria) at room temperature of 25 °C. The sample was cut into a cylindrical shape with a diameter of 25 mm. The time sweep test was performed using a flat plate model under the conditions of a frequency of 0.5 Hz, a deformation of 1% and a preset normal pressure (*P*) of 0.4 N to record the torque change data received by the material.

(1) $$F_{n}=\frac{4T}{3R},COF=\frac{F_{n}}{\bar{N}}$$

Here, *T* was the torque during the test in μN·m, *R* was the diameter of the plate rotor in mm and *F_n_* was the frictional force in *N*. $\bar{N}$ was the average value of the actual pressure during the test, in *N*.

#### 4.3.4. Characterization of Surface Antifouling

After placing the kelp sample in a petri dish with *R* = 20 cm, it was tilted at 30°. We observed the images of the kelp samples contaminated with black ink, toluene, water, peanut blended with edible oil, and Na-bentonite. Then the kelp was simply rinsed with water. We compared the photos before and after cleaning. The toluene stained with red oil O. The water stained with olive green.

#### 4.3.5. Uniaxial Tensile Test

The tensile test was tested by WSM-10 kN universal testing machine at room temperature 25 °C. The kelp samples were cut dumbbell-shaped, which was width (*d*) = 4 mm, thickness (*t*) and length (*l*) = 50 mm. The test speed was 100 mm/min. Tensile stress was defined as force per unit area:

(2) $$Stress=\frac{F}{A_{0}}$$

where the force *F* acted on the kelp, *A*_0_ was the cross-sectional area of the initial kelp sample in mm^2^, and the cross-sectional area of the initial kelp, *A*_0_ = d × t. The σ was the value of stress when the material broke.

Tensile strain was defined as the elongation per unit length, i.e.,

(3) $$Strain=\frac{∆l}{l_{0}}$$

where Δ*l* was the stretched length of the kelp and *l*_0_ was the initial length of the kelp sample in mm. The *ε* was the value of strain when the material broke. The fracture energy (*W*), the criterion of toughness, was defined as the area under the tensile stress-strain curve, namely:

(4) $$W=\int_{\varepsilon_{0}}^{\varepsilon_{t}}Stress\;\varepsilon d\varepsilon$$

*ε*_0_ = 0, *ε_t_* was the tensile fracture strain and the elastic modulus (*E*) was calculated within the initial linear range of the stress-strain curve.

#### 4.3.6. Energy Dissipation Performance Test

The energy dissipation performance of kelp samples was tested by WSM-10KN universal testing machine. The kelp was cut into dumbbell-shapes by a cutting knife. The test temperature was room temperature and the tensile speed was 100 mm/min. The dissipated energy (*Uhys*) was defined as the area enclosed by the cyclic loading-unloading curve:

(5) $$Uhys=\int_{1}^{\lambda_{max}}\left[{Stress}_{load}-{Stress}_{unload}\right]d\lambda$$

Here, the kelp sample was loaded and stretched to the maximum stretch ratio (*λ_max_*, *λ* = *ε* + 1), and then unloaded to complete a cycle, where *λ*_0_ and *λ_max_* were the initial and maximum tensile strains and stress load was the loading process; tensile stress, stress unload was the tensile stress during unloading.

#### 4.3.7. Shear Test of Trouser Samples

The tear properties of kelp were tested using the universal testing machine WSM-10KN. First, the sample was cut into a “trouser” shape with a *l* = 80 mm, *d* = 40 mm, a thickness t and an incision c = 10 mm with a cutter. The stretching speed was 20 mm/min. The tearing energy (τ) was defined as the energy (J/m^2^) required to tear the sample, namely:

(6) $$\tau =\frac{2F}{t}\times 1000$$

where *F* was the force (*N*) experienced by the sample.

#### 4.3.8. Static Contact Angle Test

Contact angles (CAs) were measured using a JC2000D1 contact angle (USA) meter at room temperature and standard atmospheric pressure. The volume of each drop was 3 μL and the “five-point fitting method” was selected to measure the contact angle; five measurements were taken, then the static contact angle was average value. The contact angles of oil and water on the surface of kelp samples were measured in air at room temperature. Toluene was used as the lipophilic liquid, the contact angle of the surface with water was measured in oil medium and the contact angle of the surface with heavy oil chloroform was measured in aqueous medium. In addition to being used as a medium during testing, deionized water was stained bright green and heavy oil chloroform (CHCl_3_) was stained with red oil O.

#### 4.3.9. Conductivity Test

The electrical conductivity (*ρ*) of the materials was measured by VICTOR and VC4090ALCR instruments (China). The formula for calculating conductivity was:

(7) $$\rho =\frac{l}{RS}$$

where *l* and *S* were the distances between adjacent electrodes and the cross-sectional area of the ionosphere and *R* was the resistance of the sample.

#### 4.3.10. Nanotriboelectricity Test

A linear motor (R-LP4, USA) with pressure control was used to provide mechanical motion input for the study of triboelectric devices. Open circuit voltage, short circuit current and transferred charge were recorded by a Keithley 6514 electrometer. Test conditions were 5 N, 2 Hz.

## Data Availability

The original contributions presented in this study are included in the article/Supplementary Materials. Further inquiries can be directed to the corresponding authors.

## Supplementary Materials

The following supporting information can be downloaded at: https://www.mdpi.com/article/10.3390/gels11080597/s1, Figure S1: (a) Changes in thickness and quality of kelp over leaching time. (b) The relationship between the thickness of kelp and the *σ* and *ε* in // directions. (c) The relationship between *σ* and leaching time of kelp. (d,e) The change of Cl^−^ concentration and COF in kelp with leaching time. Figure S2. The kelp adsorbed Ca^2+^. (a) SEM image of cross section, and (b) distribution of Ca^2+^ and (c) Cl^−^.
