# Supplementary material for: Natural Kelp (Laminaria japonica) Hydrogel with Anisotropic Mechanical Properties, Low Friction and Self-Cleaning for Triboelectric Nanogenerator
Source: Gels. 2025 Aug 1;11(8):597. doi: 10.3390/gels11080597 (PMC12385887; doi:10.3390/gels11080597)
Supplement: Supplementary file 1 [file gels-11-00597-s001.zip › gels-3747119-supplementary.pdf]

Supporting Information

Natural Kelp (Laminaria japonica) hydrogel with Anisotropic Mechanical Properties, Low Friction and Self-Cleaning for Triboelectric Nanogenerator

Dongnian Chen[1, #], Hui Yu [2, #], Hao Jiajia [2, #], Qiang Chen [2, *], Lin Zhu [3, *]

[1] Taizhou Institute of Science and Technology, Nanjing University of Science and Technology, Taizhou, Jiangshu 225300, China.

[2] Wenzhou Institute, University of Chinese Academy of Sciences, Wenzhou, Zhejiang 352001, China.

[3] Oujiang Lab, Wenzhou, Zhejiang 352001, China.

[#] Equal contribution.

* Corresponding author:

Qiang Chen, chenqiang@ucas.ac.cn

Lin Zhu, zhulin202109@163.com

1 Results and Discussion

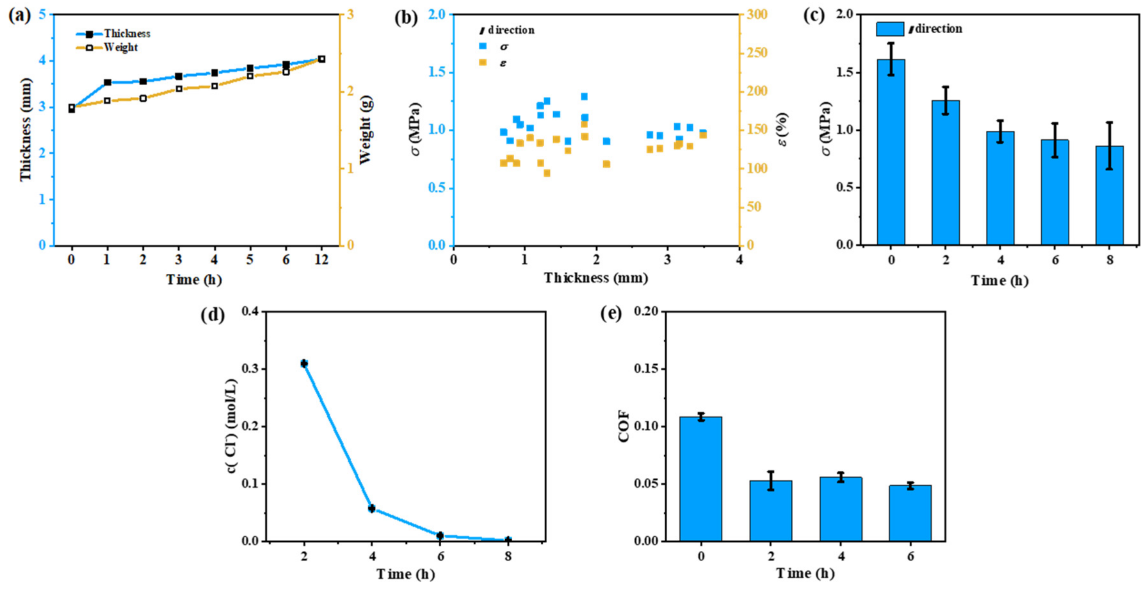

Figure S1 (a) Changes in thickness and quality of kelp over leaching time. (b) The relationship between the thickness of kelp and the σ and ε in // directions. (c) The relationship between σ and leaching time of kelp. (d-e) The change of Cl^- concentration and COF in kelp with leaching time.

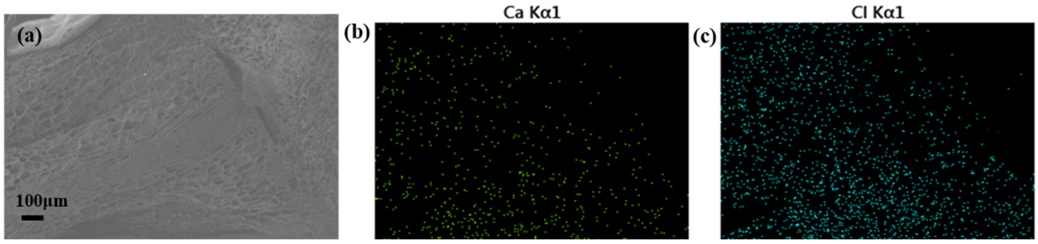

Figure S2 The kelp adsorbed Ca^{2+}. (a)SEM image of cross section, and (b) distribution of Ca^{2+} and (c) Cl^-.
